# Effective Genetic Expression of Nanoantibodies by Recombinant Adenoviral Vector in vitro

**Published:** 2011

**Authors:** I.Yu. Gribova, S.V. Tillib, I.L. Tutykhina, М.М. Shmarov, D.Yu. Logunov, L.V. Verkhovskaya, B.S. Naroditskii, A.L. Gintsburg

**Affiliations:** Gamaleya Research Institute for Epidemiology and Microbiology; Institute of Gene Biology, Russian Academy of Sciences

**Keywords:** recombinant adenoviral vector, nanoantibodies, genetic immunization

## Abstract

The present study is devoted to the feasibility of expressing the single-domain mini-antibody (nanoantibody) selected from the library of sequences of the variable domains of special single-stranded antibodies derived from an immunized camel, a gene of which was introduced into eukaryotic cells within a recombinant adenoviral vector. A vector bearing the gene of a single-domain nanoantibody was obtained using the AdEasy Adenoviral Vector System (Stratagene). This method of delivering the nanoantibody gene facilitates efficient expression of this gene and functional activity of the nanoantibody. The results obtained can be used to produce passive immunizing tools against pathogens or new-generation immunobiological antitoxic medication.

## INTRODUCTION

Antibodies are the primary tools of the immune system, which participate in the protection of the organism against pathogenic microorganisms. The significance of antibodies is growing as researchers become aware of their potential not only as tools to be used in diagnostics, but in therapy as well [1]. Antibodies have been successfully used to treat certain forms of oncological conditions. Over the past decades, monoclonal antibodies have been widely used in diagnostics and for research purposes. Yet, the conventional methods used to obtain monoclonal antibodies, based on dealing with animal-origin cells, make difficult their use as therapeutic agents. Introduction of these monoclonal bodies into the human organism may result in the onset of an undesirable immune reaction, particularly, if used repeatedly [2]. In order to prevent the emergence of such an immune response, the following approaches have been developed: production of recombinant immunoglobulins in which the regions that are not responsible for antigen recognition are replaced by corresponding fragments of human origin (humanized antibodies), or removal of the domains that are not involved in antigen binding (mini-antibodies). The so-called recombinant technologies, based on the use of libraries comprising sequences from human antibodies, have found increasing application over the past decade. When constructing these libraries, variable domains of the heavy and light strands are linked in the expression vector via random screening within one reading frame via the linker sequence [3]. It is rather laborious to deal with cumbersome libraries of these single-stranded antibodies (scFv), and only in rare cases is a highly affine antibody finally obtained. Certain difficulties are associated with the instability of genetic constructions, the low level of product expression, and its solubility [4].

A significant breakthrough in this field has been the detection of non-canonical antibodies in members of the Camelidae biological family. These antibodies do not contain light strands and represent a dimer of shortened heavy strands [5, 6]. An immune response with the participation of these antibodies can be induced by conventional immunization. There are a number of advantages in using the repertoire of these non-canonical antibodies to create libraries of sequences of variable domains (for the heavy strand only). The single-domain structure of the recognizing variable domain stipulates a small size of the antigen-binding fragment (mini-antibodies), high stability, and solubility [7].

Thanks to their structure, mini-antibodies can be used to reveal epitopes that are hidden for the conventional immunoglobulins. The expression from a single gene simplifies genetic engineering procedures and, therefore, the work with the libraries containing the sequences of variable domains. Low immunogenicity (conditioned by the high homology of the sequences of mini-antibodies with a variable domain of heavy strands of human IgG3) and the relative simplicity of the humanization procedure open broad opportunities for the application of mini-antibodies in the design of novel pharmaceutical agents [8].

These features of the structure of mini-antibodies and the simplicity with which their genes can be manipulated enable efficient and economical production of large amounts of a mini-antibody, using various expression systems [9]. 

The use of the prokaryotic expression system to produce mammalian proteins has to do with the possibly low functional activity of the proteins obtained, due to the absence of a system for post-translational modification in prokaryotic cells. Moreover, no matter how thorough the purification, the final product can still be contaminated with pyrogenes.

One of the promising methods for delivering genetic material to target cells is the use of viral vectors. Expression constructions bearing one or several recombinant genes are incorporated into the viral genome using methods of genetic engineering. Vectors based on the genome of the adeno-associated virus have been proposed in a number of studies [10, 11] for delivery of mini-antibody genes to target cells.

Adenoviral vectors are among the most universal tools used for delivery and expression of recombinant genes in mammalian cells. It is known that recombinant adenoviruses efficiently transfer the genes of bacterial and viral antigens, cytokines, growth factors, and other proteins to the target cells, ensuring a high level and duration of target gene expression [12]. Adenoviral vectors are capable of transducing both dividing and postmitotic cells. Adenoviral DNA remains in its extrachromosomal form, whereas the recombinant virus is excreted from the organism within 4–5 weeks [13, 14].

The production of recombinant adenoviruses is characterized by the following feature: the virus is capable of reproducing only * in vitro* in special cell lines, which ensures the vector’s safety [15]. 

The fact that recombinant adenoviral vectors can be used efficiently for the expression of antigen-binding fragments of antibodies is borne out by the example of mini-antibodies to the cell epitope (the epidermal growth factor receptor (erbB-2) and anthrax toxin component) [16, 17].

The aim of the present work is to examine how recombinant adenoviral vectors can be used for delivery and efficient expression of single-domain mini-antibodies (nanoantibodies) obtained using the novel technology of generation of special single-stranded antibodies extracted from camel. The nanoantibody earlier obtained and characterized to the cell cytokeratine-8 [18] was selected as the model antibody. It was subsequently used to demonstrate the fundamental possibility of expressing the single-domain antibodies obtained by immunization of members of the Camelidae family via recombinant adenoviruses.

## EXPERIMENTAL

**Enzymes**

In this study, restriction endodeoxyribonucleases, T4 DNA ligase, alkaline phosphatase (CIAP) purchased from Fermentas MBI (Lithuania), and Taq-polymerase purchased from Promega (United States) were used.

**Cell lines**

The HEK-293 cell line (human embryonic kidney cell culture transformed by the E1-region of human adenovirus serotype 5) and Н1299 cell line (human lung cancer cells) were used. The cells were cultured in a DMEM medium containing 10% of fetal bovine serum (FBS) purchased from HyClone (United States).

**Production of the cDNA clone encoding the single-domain mini-antibody (nanoantibody) which specifically recognizes the endogenous mouse cytokeratin-8**

Antibody aCyK-V _H_ H, which specifically recognizes mouse cytokeratin-8, was obtained earlier by S.V. Tillib’s research group ( Institute of Gene Biology, Moscow) in collaboration with the laboratory headed by S. Muyldermans (Vrije Universiteit Brussel) and used (via binding to the fluorescent protein sequence) to obtain fluorescent nanoantibodies (or chromobodies) aimed at demonstrating the new method for tracing antigens in a living cell. It should be noted that the aCyK-V _H_ H nanoantibody was one of the first antibodies to endogenous structural eukaryotic proteins. The first stage of its production comprised immunization of the Bactrian camel ( *Camelus bactrianus* ) with a protein extract from mouse soft tissue cells (predominantly from the liver). The subsequent selection procedure, based on the phage display method, was performed as described in the online supplement to the article [18]. The fundamental stage after selection of the most enriched antibody clones was the identification of the unknown antigen recognized by these nanoantibodies. The proteins from the nanoantibody-binding region upon Western blotting were additionally separated by electrophoresis to obtain individual products. Western blotting was then used to analyze the recognition of each product by a nanoantibody. The product recognized by a nanoantibody was identified using mass spectrometrical analysis of its trypsin hydrolysate. The resulting nanoantibody aCyK-V _H_ H recognized cytokeratin-8, a fact attested to via the immunofluorescent staining of С2С12 (mouse myoblast cell line) with these antibodies, revealing the characteristic distribution of cytokeratin intermediate filaments in the cytoplasm.

The nanoantibody aCyK-V _H_ H produced in the bacterial periplasm was modified by binding an antigen-recognizing sequence of two additional small fragments, epitope of influenza virus haemogglutinine (HA-tag) and six histidine residues (His _6_ -tag), in order to purify it and simplify its detection.

**Obtaining recombinant adenovirus**

Plasmids and the recombinant adenoviral vector were obtained using the gene of antibody to cytokeratin *aCyK-V _H_ H* . The nucleotide sequence encoding the nanoantibody was obtained by chemical synthesis in “Evrogen” JSC. The AdEasy Adenoviral Vector System (Stratagene, United States) was used in order to construct the рAd-aCyK-V _H_ H plasmid vector containing the genome of the recombinant adenovirus with E1 region deletion, and a transgene expression cassette incorporated instead of it via homologous recombination in *E. coli * cells. The recombinant adenovirus was obtained via transfection of HEK-293 cell lines with the рAd-aCyK-V _H_ H plasmid construct linearized on the PacI site. Lipofectamine 2000 (Invitrogen, United States) was used for the transfection, according to the manufacturer’s recommendations. The recombinant human adenovirus of serotype 5 with E1 region deletion and an incorporated transgene-free cassette expression (Ad-null) inserted instead of it was used as the control.

To accumulate adenoviral preparations, an infected cell suspension (10 ^7^ PFU of the virus per Petri dish with a diameter of 15 cm) was coated to the HEK-293 cell monolayer with 50–70% confluence. The infected cell suspension was destroyed by three freeze-thaw cycles and clarified by centrifuging (2000 rpm, 10 min, +4°С).

The titres of the specimens Ad5-aCyK-V _H_ H and Ad-null (10 ^8^ PFU/ml) were determined by the plaque formation technique in the HEK-293 cell culture.

**Infection of cells with  a recombinant adenovirus**

Approximately 10 ^6 ^ cells of the H1299 line were infected with recombinant adenoviruses. The cells were seeded to ~ 70% of the monolayer, cultivated for 24 h, and infected with the recombinant adenovirus (the multiplicity of infection being 100 PFU/cell) in a DMEM medium containing 2% of FBS. Two hours after the viral preparation was introduced, the medium was collected, the cell culture was washed, and a fresh DMEM medium was added. The medium from the infected cells was collected 72 h after infection and concentrated by centrifuge ultrafiltration through a membrane with a nominally intercepted molecular weight of 10 kDa. After thickening by a factor of 10, the supernatant was fractioned in a 10% polyamide gel and used for immune blotting analysis.

**Antigen preparation**

Homogenized mouse liver lysate (BALB/c line) was obtained via extraction with the use of a RIPA buffer (50 mM Tris-HCl, pH 8.0, 150 mM NaCl, 1% NP-40, 0.5% sodium deoxycholate, protease inhibitor kit (Roche, Switzerland)). The concentration of the total protein in the specimens was determined by the Bradford method (Sigma-Aldrich, United States). The specimens with an equal protein concentration were applied to the gel to be separated by electrophoresis.

**Polyacrylamide gel electrophoresis and immunoblotting**

Cellular proteins were separated by polyacrylamide gel electrophoresis by the Laemmli procedure under denaturing conditions in the presence of sodium dodecyl sulphate. Protein Test Mixture 4 (Serva, Germany) was used as the molecular weight standard. After the gel electrophoresis, the proteins were placed onto a Hybond-P PVDF membrane (GE Healthcare, United States) using a TE70 Semi-Dry Transfer Unit (Hoefer Scientific, United States) in accordance with the manufacturer’s recommendations. The nanoantibodies were detected using the Monoclonal Anti-HA–Peroxidase antibody (Sigma-Aldrich, United States). The immobilized proteins were detected using ECL Plus Western Blotting Detection Reagents (GE Healthcare, United States) in accordance with the manufacturer’s recommendations. The chemilumenscent radiation was recorded with the aid of an Amersham Hyperfilm ECL X-ray film (GE Healthcare, United States).

## RESULTS

As a result of the earlier performed selection of the phage library of the antigen-binding domains of single-stranded antibodies, DNA from the pHEN4 phagemid with an insertion encoding the nanoantibody, which has a high affinity towards the structural cytoplasmatic mouse protein, cytokeratin-8, was collected. The data on the structure of the target protein were obtained via mass spectrometric identification. The nucleotide sequence encoding the nanoantibody was cloned in the recombinant adenoviral vector.

**Fig. 1:**
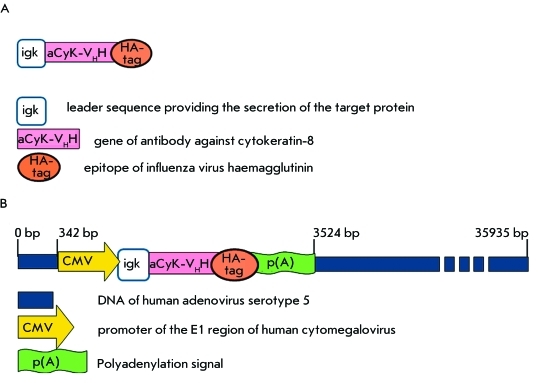
Schematic description of the genetic constructions used. A – Genetic construction containing the gene of nanoantibody against cytokeratin. B – The recombinant adenovirus genome bearing the gene of nanoantibody against cytokeratin.

A leading peptide of the mouse immunoglobulin κ-chain was bound to its N-terminus, in order to ensure efficient extracellular expression of a nanoantibody. The HA-tag, which is effectively recognized by commercial antibodies, a requirement for confirming nanoantigen expression at the protein level, was bound to the C-terminus of the nanoantibody. *Fig. 1* shows the scheme of the resulting construct.

In order to construct the adenoviral vector, the sequence encoding the HA-tag-labelled аCyK-V _H_ H antibody was cloned in the shuttle plasmid vector pShuttle-CMV (Stratagene, United States). This vector contains terminal fragments of the human adenovirus serotype 5 genome, the expression cassette containing the human cytomegalovirus promoter (CMV) and polyadenylation signal. The presence of the insertion and its orientation were confirmed by restriction mapping.

The recombinant plasmid adenoviral vector bearing the target gene was obtained via homologous recombination in *E. coli * cells. The plasmid construct obtained contained the replication initiation site ori, the gene of antibody resistance, and a cassette with the target gene within the adenoviral genome. The main advantage of this method is the potential utilization of *E. coli* cells as the main tools for cloning, recombination, and production of adenoviral DNA in preparative amounts. The opportunity to perform the homologous recombination in *E. coli * cells makes it possible to deal with the individual clones containing plasmid constructs only with recombinant adenoviruses, which eliminates the possibility of contamination with a wild-type adenovirus.

The shuttle plasmid construct bearing the expression cassettes with the nanoantibody gene was linearized on the PmeI site and introduced along with pAd-EASY (Stratagene) to *E. coli* BJ5183 cells by electroporation. Recombinant clones obtained by homologous recombination were collected on the selective kanamycin-containing medium (50 µg/ml). The presence of recombinant clones of nucleotide sequences encoding the aCyK-V _H_ H antibody and human adenovirus serotype 5 fibre in plasmid DNA was analyzed by PCR with specific primers and via restriction mapping, using HindIII restrictase, which enables one to obtain a restriction pattern that is typical for the human adenovirus genome.

HEK-293 cells were transfected with a plasmid cleaved at the PacI site and containing the recombinant adenovirus genome with E1 region deletion and the expression cassette with a transgene inserted instead of it. The resulting recombinant adenovirus Ad5-aCyK-V _H_ H was analyzed by PCR using the primer pair that was complementary to the target gene, the hexon gene of human adenovirus serotype 5, and the E1 region of the adenovirus in order to control the possible presence of replication-competent viral particles. 

**Detection of the expression of the  nanoantibody gene within the recombinant adenovirus Ad5-aCyK-V _H_ H**

**Fig. 2:**
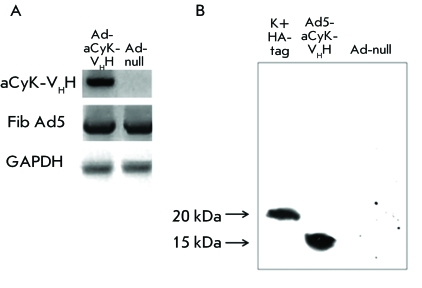
The analysis of anti-cytokeratin nanoantibody expression in cells infected with recombinant adenovirus. A – Expression of anti-cytokeratin nanoantibody gene in cells infected with recombinant adenovirus Ad5-aCyK-VHH was analyzed using reverse transcription (RT)-PCR, cDNA encoding this gene was amplified by PCR with primers specific to the gene of the anti-cytokeratin nanoantibody (aCyK-VHH), Ad5 fiber gene (Fib Ad5), and house-keeping gene GAPDH. A recombinant adenovirus with no transgenic insertions in the E1 deletion region of the adenoviral genome (Ad-null) was used as the specificity control. B – The expression of the anti-cytokeratin nanoantibody was detected by hybridization with anti-HA antibodies in a Western blot analysis. A protein with a molecular weight of 15 kDa was detected in the cultural fluid of cells infected with the recombinant adenovirus. The (His _6_ )-tagged nanoantibody produced in E. coli was used as the control of the specificity of the interaction between anti-HA antibodies and the target protein.

The expression of the target gene within the recombinant human adenovirus serotype 5 Ad5-aCyK-V _H_ H was analyzed at the level of the mRNA. With this purpose in mind, the cells of the HEK-293 line that are permissive for human adenovirus serotype 5 were infected with the recombinant virus Ad5-aCyK-V _H_ H. The total RNA of infected cells was used to produce DNA, which was analyzed by PCR with primers specific to the sequence of the nanoantibody gene to mouse cytokeratin 8, to viral DNA, and the constitutively expressed gene of glyceraldehyde-3-phosphate dehydrogenase (GAPDH). HEK-293 cell lines infected with Ad-null virus ( *Fig. 2A* ) were used as the negative control. RT PCR was used to demonstrate that the recombinant adenoviral vector expresses mRNA of the nanoantibody gene to cytokeratin and can be used to analyze protein production.

Nanoantibody expression at the translational level was analyzed in H1299 cells infected with the recombinant adenovirus carrying the gene of nanoantibody to aCyK-V _H_ H tagged with HA-epitope of the influenza virus (Ad5-aCyK-V _H_ H), and the recombinant adenovirus containing the transgene-free expression cassette (Ad-null). The presence of a nanoantibody in the culture medium containing the infected cells was measured by immunoblotting with antibodies to HA-epitope of the influenza virus conjugated with horseradish peroxidase ( *Fig. 2B* ).

**Biological activity**

The specificity of a nanoantibody expressed by the adenoviral vector to cytokeratin was confirmed by means of comparison of the interaction between the antigene and the proteins of the cultural fluid from the cells infected with recombinant adenovirus, and the interaction between the antigene and the antibody purified from *E. coli * periplasm. 

The lysates of mouse liver and cerebrum cells were fractioned in a polyacrylamide gel, transferred to the PVDF membrane, which was incubated with the cultural medium of the cells infected with Ad5-aCyK-V _H_ H. The expressed nanoantibody served as the primary antibody to the target protein (mouse cytokeratin-8, 55 kDa) detected in the total lysate. The membrane was simultaneously incubated with antibodies aCyK-V _H_ H produced in *E. coli* periplasm.

**Fig. 3:**
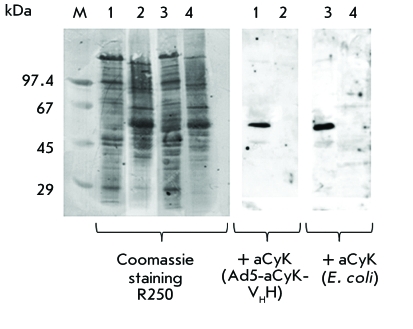
Western blot detection of the functional activity of nanoantibodies. Total cell extracts of liver (lanes 1, 3) and brain (lanes 2, 4) cells were separated on a SDS–PAGE and electrophoretically transferred to a PVDF-membrane. The specific interaction of the target protein (~ 55 kDa) with the anti-cytokeratin nanoantibodies obtained in the cultural fluid of cells infected with the recombinant adenovirus and periplasm of *E. coli* was detected by immunoblotting.

*Fig. 3* shows the results of an electrophoresis of protein lysates in polyacrylamide gel and the data obtained by immunoblotting with nanoantibodies aCyK-V _H_ H after development by secondary antibodies to the HA-epitope of the influenza virus conjugated with horseradish peroxidase.

Immunoblotting results attest to the fact that the antibody expressed by the adenovirus has the same specificity as the antibody synthesized in *E. coli* periplasm, its gene being cloned in the recombinant adenovirus.

## DISCUSSION

At the time of writing, there were a number of technologies capable of producing mini-antibodies with a predetermined specificity. Only quite recently was it revealed that, in addition to the canonical antibodies, functionally active noncanonical single-stranded antibodies were produced in relatively large amounts in members of the Camelidae family. Therefore, it is now possible to obtain mini-antibodies on the basis of libraries containing the antigen-recognizing domains of single-stranded antibodies of immunized animals. Noncanonical antibodies consist of a dimer with a single shortened heavy immunoglobulin chain (containing no light chains). Single-domain mini-antibodies (nanoantibodies) are genetically engineered derivatives of the antigen-recognizing domains of these noncanonical antibodies. The selection of clones of a mini-antibody with the predetermined specificity from the library of sequences of the entire repertoire of antigene-recognizing domains of noncanonical antibodies obtained from immunized camel is based on the highly efficient procedure of functional selection of filamentous phage particles containing both an exposed mini-antibody on the surface, and the DNA encoding it within the phage particle (phage display).

Mini-antibodies produced by this technology are characterized by high stability, solubility, and low immunogenicity. Mini-antibodies can be produced (selected) to any antigens and any antigen epitopes, including conservative ones, which often cannot be produced using the conventional procedure. Since the encoding nucleotide sequence is known for each mini-antibody, it is possible to produce the corresponding protein in any of the known expression systems (prokaryotic and eukaryotic).

It is economically viable to produce protein preparations of mini-antibodies in *E. coli * cells, yeast, or CHO cells. When injecting these preparations to experimental animals (or patients), their very short lifetime in the organism (less than 24 h) should be taken into account. The period of therapeutic action of preparations based on mini-antibodies can be increased using the vector systems, providing that the synthesis of the active agent takes place immediately in the infected cells of the organism. Recombinant adenoviruses are the optimal expression system for solving such problems. Their safety and efficiency has been proved in a number of clinical trials performed globally; the time needed to produce a target protein is approximately 20 days. 

The potential application of recombinant adenoviral vectors for the expression of the genes of the antigen-recognizing fragments of single-stranded antibodies obtained from Bactrian camel was studied in this work. It was demonstrated that expression of the nanoantibody gene using the adenoviral vector is possible. Transgene expression was confirmed at the level of the RNA transcript and protein product. The specific interaction of the nanoantibody secreted by eukaryotic cells with a target protein attests to the fact that its functional activity is retained. Further studies are necessary for a qualitative estimation of the efficiency of nanoantibody expression using a recombinant adenovirus.

## CONCLUSIONS

The delivery of the gene of a single-domain mini-antibody (nanoantibody) selected from the library containing sequences of the variable domains of specific single-stranded antibodies of immunized camel to eukaryotic cells using the recombinant adenoviral vector provides efficient expression and functioning of the nanoantibody. The results of this study can be used for the production of passive immunization agents for protection against pathogens, or for the design of new-generation immunobiological antitoxic preparations. 

## Abbreviations

HEK-293: human embryonic kidney cell culture
His_6_-tag - amino acid motif in proteins consisting of six histidines; НА-tag: epitope tag (YPYDVPDYA) derived from the haemagglutinin molecule
PFU: plaque-forming unit
PCR: polymerase chain reaction
PT-PCR: reverse transcription polymerase chain reaction
